# Exploring Lay Understandings of Romantic Chemistry Using Inductive and Deductive Content Analysis

**DOI:** 10.3390/bs15111565

**Published:** 2025-11-17

**Authors:** Scott Devenport, Matthew J. Phillips, Barbara Mullan, Sam Winter, Catriona Davis-McCabe

**Affiliations:** School of Population Health, Curtin University, Bentley, WA 6102, Australia; matthew.phillips@curtin.edu.au (M.J.P.);

**Keywords:** romantic chemistry, lay understanding, content analysis, romantic partner selection, early relationship formation

## Abstract

Romantic chemistry is an important indicator of compatibility between prospective romantic partners, but, despite theoretical work, lay understandings of romantic chemistry that could inform theory are still unclear. We used an online survey question to collect romantic chemistry conceptualisations from 571 Australian adults who were currently looking for a romantic partner, of whom 53.06% identified with minority gender and/or sexual identities. We analysed responses using inductive content analysis, which resulted in the construction of categories and sub-categories concerning the multifaceted nature of romantic chemistry, the importance of mutual feelings, and central concepts of interactivity, connection, and attraction. We performed a deductive content analysis using these categories and sub-categories to re-code responses and observed little evidence of differences between groups based on assigned sex, gender, sexual, and minority identities. Our findings suggest that romantic chemistry is only perceivable when multiple facets are experienced and that experiences of facets vary individually, which provides ample grounds for future investigation and measurement of romantic chemistry.

## 1. Introduction

Romantic chemistry has remained a nebulous concept despite being central to lay and academic understandings of romantic experiences (Eastwick et al., 2007; Reis et al., 2022). One explanation for this could be that individuals conceptualise interpersonal chemistry differently and further differentiate romantic chemistry from other forms of interpersonal chemistry such as those between friends or teammates (Campbell et al., 2018). This differential conceptualisation is reflected in research where definitions of romantic chemistry include compatibility (Baxter et al., 2022), sexual desire (Simons et al., 2024), and event-specific feelings such as those around a first kiss between prospective romantic partners (Thompson et al., 2023). Romantic chemistry can be conceptualised as containing some or all these aspects depending on the individuals and context they are in (Reis et al., 2022), but this complexity is sometimes ignored in research (Simons et al., 2024) and may not enter lay understanding (Shen & Qian, 2024). While theoretical work on romantic chemistry has been undertaken (Campbell et al., 2018; Reis et al., 2022), there remains scope to further our understanding of this complex phenomenon.

Romantic chemistry typically involves intense positive feelings such as passion or sexual attraction that drive relatively quick, intense progress towards perceptions of chemistry (Reis et al., 2022). The view that romantic chemistry is associated with a quick, almost instant connection is frequently seen in lay understandings (Shen & Qian, 2024); however, deeper connections and compatibility that may take longer to eventuate appear to also contribute towards conceptualisations of romantic chemistry (Baxter et al., 2022; Cai & Qian, 2023). Longer-term elements of romantic chemistry, including shared emotions and consistent appropriate responsiveness to partners, have been associated with increased relationship satisfaction, further supporting the importance of these elements (Brown et al., 2022; Tou et al., 2018). Despite both short- and long-term elements of romantic chemistry being reportedly experienced, researchers have more often focused on instantaneous or first contact romantic chemistry and perhaps overlooked the complexity introduced by longer term elements (Campbell et al., 2018; Reis et al., 2022).

Reis et al. (2022) developed a theoretical framework of interpersonal chemistry, and argue that chemistry is largely a felt phenomenon, a lived experience, which is perceived by individuals after repeated moments of connection. These moments of connection arise from interactivity between individuals; an individual’s behaviour is met with a response from another individual and positive evaluations of these moments (i.e., connections) may lead to perceptions of chemistry. These perceptions of chemistry could require multiple moments of connection, intensify over repeated moments, or occur from the first moment. Importantly, these moments of connection must result in specific cognitive, affective, and behavioural outcomes; cognitively, a perception of shared identity with another individual such as similar interests, affectively, positivity towards another individual such as attraction, and behaviourally, a perceived coordination on activities relevant to goals of one or both individuals, such as mutual interest in a date. Together, these outcomes contribute to the overall perception of chemistry, which can be reinforced by subsequent moments of connection through continuing interactivity and positive evaluations.

The broad nature of romantic chemistry allows for connections to broader theories of love (Sorokowski et al., 2021), attachment (Hazan & Shaver, 1987), and other evolutionary processes such as desire (Fisher et al., 2002). Evolutionary processes of love and attachment processes are likely to be influential, but may not be as dominant in the early stages of relationship formation such as dating (van Anders, 2015). Indeed, love and attachment may be more appropriately framed as longer-term elements of romantic chemistry, but the shorter-term elements of lust and desire may be more applicable to dating contexts (Reis et al., 2022). There is also some limited evidence that perceptions of romantic chemistry may better predict early relationship formation determinants such as reciprocal liking (Eastwick et al., 2007). The connections to shorter- and longer-term contexts and various phases of romantic relationships make the romantic status of the population being studied important, especially when considering the highly subjective nature of romantic chemistry perceptions. Unfortunately, the status of samples used in previous research is not always provided, which makes further determinations in this direction difficult (Campbell et al., 2018).

Despite the experiential nature of interpersonal chemistry creating ample scope for qualitative investigations, Campbell et al. (2018) represents one of few qualitative investigations in this field. Campbell and colleagues inductively analysed data from open-ended survey questions asking about friendship and romantic chemistry, from which they constructed the following eight themes, presented in order of most frequently coded for romantic chemistry: ‘Reciprocal Candor’, ‘Mutual Enjoyment’, ‘Attraction’, ‘Similarities’, ‘Personableness’, ‘Love’, ‘Instant Connection’, and ‘Indescribable’. ‘Attraction’ and ‘Love’ themes were more frequently coded within romantic chemistry responses in comparison to friendship chemistry responses, whereas the ‘Similarities’ theme was less frequently coded. This finding indicates that attraction and powerful feelings such as love may be the features that distinguish romantic chemistry from other forms of interpersonal chemistry. The eight themes identified by the authors mostly align with the theoretical category of affective positivity proposed by Reis et al. (2022), with categories of perception of shared identity and perceived coordinated goal-relevant activity less aligned—and perhaps less relevant to romantic chemistry as the ‘Similarities’ theme was less frequently coded.

Campbell et al. (2018) provides useful, necessary qualitative insights into the romantic chemistry phenomenon, but there are some problems with the study that limit the interpretability of their findings. It is unclear what qualitative methodology the authors used as they list only the constant comparative method that is usually employed within a grounded theory methodology, but grounded theory is not mentioned, nor is any other methodological framework. This lack of clarity raises questions about how themes were constructed, even when the authors report sound quality practices. Some of the authors’ decisions appear unjustified, such as coding responses from men and women separately; while the study did plan to compare responses between men and women, it is unclear why the researchers needed to separate, and potentially have knowledge of, the gender associated with each response, and this could have biased analyses. The lead author’s previous work on friendship chemistry is cited but the overlap between the quantitative dimensions observed in that study (Campbell et al., 2015) and the inductive themes of the Campbell et al. (2018) study is not discussed with enough transparency to assuage concerns about the potential for bias in their inductive analyses. The authors also define interpersonal chemistry as “a perceived connection with a person that is evident on first meeting” (Campbell et al., 2018, p. 34), pre-empting, and perhaps preventing, any analytical outcomes related to long-term elements of chemistry. Despite the utility of Campbell and colleagues’ study, these issues raise concerns about the quality practices of the study and create a need for further qualitative investigations to replicate and expand upon the authors’ findings.

There is emerging theory about the nature and function of romantic chemistry, and research has begun to test these theories. As romantic chemistry is a subjective phenomenon, it is difficult to capture quantitatively despite being evidentially important (Eastwick et al., 2007). Romantic chemistry is also a phenomenon largely understood through ambiguous lay definitions; therefore, qualitative investigations are a suitable starting point to expands our academic understanding and resolve ambiguity. Unfortunately, previous qualitative research on romantic chemistry has either been narrow in focus or definition regarding the nature of romantic chemistry, or methodologically questionable. Furthermore, previous studies have sampled mostly from cisgender and heterosexual populations (Campbell et al., 2018); this may have omitted potentially illuminating contributions from those of gender and sexual identities in the minority who sometimes report differing romantic experiences due to their identity (Albury et al., 2021; Devenport et al., 2025; Vares, 2018).

In our current study, we aim to address the conceptual, methodological, and sampling concerns identified in the literature by qualitatively exploring how a diverse group of individuals conceptualise romantic chemistry. A diverse sample enables exploration of how gender and sexual identities may influence conceptualisations of chemistry and enhances the applicability of study findings, while a sample of participants that are actively in the mindset of desiring romantic experience may provide further insights specific to this early relationship formation context. Ultimately, our study will address the research question: how do individuals of diverse gender and sexual identities currently looking for a romantic partner experience and conceptualise romantic chemistry, and what similarities and differences are observable between identity groups?

## 2. Method

### 2.1. Research Design and Positionality

Our study was exploratory, and we analysed qualitative data from an open-ended survey question using inductive and deductive content analyses. We selected content analysis due to the higher likelihood that survey responses produce data that are more descriptive than rich in meaning, preventing deeper qualitative analyses (Kyngäs et al., 2019; Vears & Gillam, 2022). We adopted a social constructionist epistemology as we agree with previous research that suggests that romantic chemistry is experienced, but in a manner that can be shared between individuals and is part of social knowledge, which results in a social construction of the experience and conceptualisations of the phenomena (Campbell et al., 2018; Reis et al., 2022). The research team holds various positions in terms of gender and sexual identities which are linked to romantic experiences, and these varying positions were leveraged to discuss potential biases and enhance the analytical process. As [first author] performed all primary analyses, it is important to acknowledge their position as a straight, cisgender man of European descent and the potential for this position to influence the research due to pre-formed Western, potentially heteronormative conceptualisations of romance and chemistry. The first author also acknowledges that being married for multiple years and therefore distanced from dating experiences may bias their interpretations toward relationship-relevant romantic chemistry concepts. The research team regularly discussed the potential biases related to the positionality of the first author and the broader team, with any indications of bias considered and managed to the best of our knowledge.

### 2.2. Participants

We used convenience sampling to recruit participants who were Australian adults currently searching for a romantic partner, as we expected this status would allow participants to draw from more recent experiences of romantic chemistry. Recruitment took place online in early 2021 via social media posts in dating, minority gender and sexual identities pages, and general Australian pages (e.g., dating-related Facebook groups, Reddit subreddits, university-related webpages). Of the 853 respondents to a larger survey, 571 participants formed the final sample by responding to the open-ended question about romantic chemistry and completing all demographic information. The final sample had an average age of 32.02 years (*SD* = 10.10), with 59.54% (*n* = 340) of participants being assigned female at birth and one participant identifying as intersex. The majority (77.06%) of participants’ ethnicities were of European descent with 7.01% of Asian descent, 5.95% of mixed ethnicity, and other ethnicities less than 1% each. Approximately half of the final sample identified with majority gender and sexual identities (i.e., cisgender and heterosexual, 46.94%) and the details of minority gender and sexual identity groups are reported in Table 1.

### 2.3. Procedure and Materials

We received ethical approval from the Curtin University Human Research Ethics Committee (HRE2021-0065). Data were collected from an open-ended question, “In romantic situations, what is ‘chemistry’ to you?”, as part of a larger survey on romantic partner selection experiences. The open-ended question was presented early in the survey, after demographic questions, and after some single-item experience questions unrelated to this study. Participants were not forced to respond to demographic or open-ended questions and there was no restriction on the length of open-ended responses. Overall, the larger survey took approximately 20 min to complete.

### 2.4. Data Analysis and Quality Procedures

Our content analysis approaches were informed by Kyngäs et al. (2019) and Vears and Gillam (2022). Analysis was performed using NVivo 14 (Lumivero, 2023). Responses were coded by the first author in an iterative process where codes and a codebook were developed and organised into structures based on response content, shared meaning, and frequency. These coding structures were reassessed and reorganised iteratively during the analysis process. Codebooks were reviewed by the fourth and last authors at multiple points throughout this process to enhance the quality of the analysis. Reflective practice was an important part of this process that was enabled by the first author keeping a reflexive journal and regularly discussing the analysis and findings throughout the coding process with the fourth and last authors (Bengtsson, 2016; Erlingsson & Brysiewicz, 2017). These processes resulted in a preliminary set of categories and sub-categories that reflected participants’ conceptualisations of romantic chemistry.

The preliminary category structure was used for an intercoder process whereby the first author, the second author, and a third-party researcher outside the research project deductively coded responses into categories and subcategories to test intercoder reliability and evaluate the inductively constructed categories (Kyngäs et al., 2019; O’Connor & Joffe, 2020). The second author and the third party were unfamiliar with the data prior to this intercoder process. Responses could be coded into more than one category, but when a category had sub-categories, a response was only coded to the main category if it contained elements that were either general to the category or ambiguous. The first round of deductive analysis used 50 randomly selected responses and resulted in moderate intercoder reliability (average Fleiss’ Kappa = 0.45). The category structure was altered slightly following discussions between coders, and the second round of deductive analysis using 25 different randomly selected responses resulted in a slight increase in intercoder reliability (average Fleiss’ Kappa = 0.50). A third round of coding was undertaken with a further 50 random responses after categories were further refined, but intercoder reliability remained moderate (average Fleiss’ Kappa = 0.50). Although stronger intercoder reliability would have been ideal, the differences in interpretation were discussed by the coders and deemed to be minor and understandable given the nebulous nature of romantic chemistry (O’Connor & Joffe, 2020).

Due to the moderate intercoder reliability, it was decided that the first author would use the finalised categories and knowledge gained through the intercoder process to deductively code all responses. The second author then reviewed this final coding and discussed any potential issues with the first author. Only minor amendments resulted from the final coding review and discussion. After this co-validation process, the final deductive coding of the entire dataset was deemed appropriate.

## 3. Findings

Our inductive content analyses resulted in the construction of five categories and four sub-categories described in Table 2, alongside proportions of each category and sub-category. The categories represent broad and distinct patterns in participants’ conceptualisations of romantic chemistry. The sub-categories represent specific and distinct patterns of responses within categories relating to connection and attraction. We detail each category and sub-category within this section before detailing our further deductive analyses. Our interpretations and descriptions of each category and sub-category are supported by the quotes presented in Table 3. As there were sub-categories for connection and attraction, we calculated the number of unique responses coded across these category sets; the connection set was coded in 48.51% of responses and the attraction set was coded in 57.09% of responses.

### 3.1. Multifaceted or Complicated

Approximately half of participants conceptualised romantic chemistry as being multifaceted by listing aspects or implying that multiple simultaneous aspects were required to qualify an occurrence as romantic chemistry. While some participants seemed confident in their understanding, others appeared to find the multifaceted nature less tangible, for example: “Don’t know how to describe it. Often based off banter and humour for me initially. Senses: touch, taste, smell… pheromones?” The complications introduced by this multifaceted nature sometimes prevented complete understanding of the experience, but it was special and correct: “Something you’ve never felt but you just know it’s right.” It was clear that many participants felt that romantic chemistry required a combination of interactivity, connection, and attraction which are broadly covered by the other categories. When this combination of factors was experienced, it was a simultaneously confusing, wonderful, and engaging experience.

### 3.2. The Importance of Mutual Feelings

Although many participants focused on their own perceptions and experiences of romantic chemistry, approximately one fifth of participants mentioned the importance of any romantic chemistry-related feelings being felt by both parties. Responses coded into this category either plainly paired the word “mutual” with other keywords such as “attraction” and “connection” or were less direct by using terms such as “each other” or mentioning the need for reciprocity. Mutual feelings were also related to equality by some participants, “Chemistry is the mutual state of feeling equally important, equally valued, and embracing non-judgmental vulnerability.” It is important to highlight that the remaining responses seemed to conceptualise romantic chemistry as occurring from their perspective only, but this may not have been the case. The survey question could have been interpreted as regarding only the individual or participants may have felt that interactivity and connection implied mutuality. Either option could explain the lower proportion of responses in this category, although it is also possible that some participants found romantic chemistry to be an individually experienced phenomenon.

### 3.3. Comfortable and Authentic Interactivity

Over half of participants’ responses focused on factors relating to easier interactivity with prospective romantic partners. Comfortable interactions felt natural and consuming, which evoked expressions of “flow” and blissful disconnection: “The feeling you can talk to someone for hours, about anything, and feel yourself losing track of time.” This comfort also allowed participants to present themselves authentically: “Being comfortable ‘being yourself’ and enjoying your partner being themselves.” This combination of comfort and authenticity during exchanges promoted feelings of safety and permitted participants to be open and vulnerable. Conversation was the primary form of interaction mentioned by participants, notwithstanding participants’ frequent addition of “being comfortable in silence” or similar. Lastly, humour and laughter were a prominent feature of this category that appeared to contribute to perceptions of comfort and authenticity, and some participants found these factors to be a requirement of romantic chemistry.

### 3.4. Feelings of Connection and Alignment

A quarter of participants highlighted general feelings of connection and alignment as an aspect of romantic chemistry, with nearly half of participant responses being coded into this category and associated sub-categories. Similarities were the most basic form of connection between parties and provided common grounds for interactivity and understanding of one another. Differences were sometimes appreciated alongside similarities, but this was not as frequently expressed by participants. The colloquial term ‘click’ was a common expression of connection that required alignment and indicated potential compatibility; while this could be interpreted as friendship ‘clicking,’ the research context implies romantic ‘clicking’. These ideas were commonly paired with those matching comfortable and authentic interactivity due to feeling connected reinforcing those benefits. Alignment in terms of “life goals”, “outlook”, or “love languages” was distinct to this category as these expressions of connection were not necessarily energetic, instantaneous, deep, or emotional. These different forms of connection and alignment highlighted how romantic chemistry could be quick and noticeable, or slower and discoverable, with both manifestations being valid from participants’ perspectives.

### 3.5. Energetic and Instantaneous Connections

Quick and noticeable forms of connection were conceptualised by some participants as energetic in origin and instantaneous in effect. This concept was frequently expressed with the terms “spark” and “electricity”, with some instead using phrases such as “magnetic attraction” to describe attraction brought on by this energy: “Mutual attractive energy between two individuals that draw them together.” Some responses coded into this sub-category included terms such as “vibe” and “wavelength” to describe energetic connections; however, not all instances of these terms were coded in this way, as some responses used them to discuss general forms of connection or comfortable interactivity. The instantaneous element of this sub-category captures participants’ experiences of quickly or “spontaneously” becoming aware of a connection to a prospective romantic partner. Participants outlined how this quick and energetic nature was “undeniable”, which made behaviour “uncontrollable” for some: “[It’s] hard to define, it’s an animal response.” Regardless of this lack of control, the energy facilitated connection, interactivity, and attraction, and was an essential part of romantic chemistry for many participants.

### 3.6. Deep and Emotional Connections

Forms of connection that required discovery of a prospective romantic partners’ personality, beliefs, and emotional compatibility were the focus of romantic chemistry for some participants. Depth was a recurring aspect of these conceptualisations that could manifest as deeply held feelings, strong connections to individuals, or intimate understanding between parties. These deeper aspects made the connection between parties more meaningful, which contributed to perceptions of intimacy and romance. Emotion was another aspect of this intimacy, and emotional connections were associated with vulnerability: “Having highly vulnerable and emotionally intelligent conversations. Spending quality time and having similar ethical values.” The term “empathy” was also used to represent emotional connections and other participants described this as “…demonstrating care for each other.” Participants’ responses implied that depth and emotional connection were slower forms of romantic chemistry, as one participant reported when contrasting instantaneous and slower forms: “A zing that extends past the shiny stage of a relationship, grows into a contentment as you go into the honeymoon stage, and then glows with love as you walk through life together.” Acknowledgement of both speeds of connection was uncommon, which may indicate that participants conceptualised romantic chemistry as occurring at only one speed.

### 3.7. Experiences and Consequences of Attraction and Interest

More than one third of participants mentioned experiences and consequences relating to attraction or similar constructs as an aspect of romantic chemistry, with more than half of responses being coded into this category and associated sub-categories. Attraction was reported in numerous forms including “physical”, “emotional”, “mental”, “intellectual”, and “spiritual”, but was also sometimes reported as simply “attraction” or as a prospective romantic partner being “attractive”. While the term “attraction” was sometimes as nebulous as romantic chemistry itself, it appeared to manifest as being “drawn to” individuals and a desire for more of a prospective partner in terms of “continued interaction” or an “insatiable urge to know someone more”. Interest was an important aspect of these experiences and was about genuinely engaging with interactions: “When there is genuine interest in each other’s stories (e.g., asking questions and responding to answers to questions genuinely and openly).” Some experiences seemed intense enough to be classified as captivation and were frequently accompanied by consequences: “Butterflies in your tummy. Unable to stop thinking about them, wanting to spend all your time getting to know them., heart racing when touching, even innocuously.” To some participants, experiences of attraction needed to be unambiguous, “Secure attraction where both feel it and don’t need to wonder or ask”, which may be due to attraction being necessary for romantic chemistry, “Chemistry is at its core a sense of attraction that draws you together.” Overall, experiences of attraction were a key component of many participants’ conceptualisations of romantic chemistry, even when the term was not clearly defined.

### 3.8. Experiencing and Expressing Physical and Sexual Attraction

A quarter of participants’ responses contained expressions that indicated their experience of attraction was at least partially of a physical or sexual nature. A simple form of this attraction was a desire for physical closeness to a prospective romantic partner. Physical attraction was also sensual, with simple touch being enough to arouse bodily reactions in many participants and sight, smell, sound possessing similar qualities. Participants also described expressing their attraction indirectly through “constant eye contact”, and directly, by holding hands or kissing to indicate or test romantic chemistry with a prospective romantic partner. Sexual attraction described the desire for sexual relations and was an expression of intimacy that was an important aspect of romantic chemistry for many. Sexual relations were also necessary to test compatibility in sexual contexts, or “sexual chemistry”, which was frequently just as important as the sexual relations. While many participants mentioned some form of physical or sexual attraction, these experiences and expressions were also frequently accompanied by aspects of interactivity and connection, indicating that these elements alone were often not enough to qualify as romantic chemistry.

### 3.9. Experiencing Intellectual Attraction

Intellectual attraction was not as commonly mentioned as other forms of attraction but was distinct and appeared to be important to some participants. Some descriptions of intellectual attraction did not expand on the idea or distinguish it from “mental attraction” or similar. Those who did expand on the idea described a “meeting of minds” and expressed enjoyment at having their thoughts and beliefs challenged by a prospective romantic partner. These challenges were linked to personal growth, which formed an important part of romantic chemistry for some participants. Shared humour communicated a common understanding and that promoted intellectual attraction for some participants, beyond simply enabling interaction and promoting connection: “A naturally occurring energy of being drawn to someone and a connection through intellect, humour, and conversations.” For the participants that found intellectual attraction to be an aspect of romantic chemistry, it was an essential element.

### 3.10. Deductive Analyses

We used Chi Square tests to compare the proportion of responses coded into each category and sub-category between participant subsamples based on assigned sex at birth (male and female) and based on gender, sexual, and majority-minority identity groups. As there were nine category or subcategory comparisons per identity grouping, the alpha level for these tests was adjusted accordingly (alpha = 0.006). Only one comparison was significant at the adjusted alpha level with responses from female participants coded more frequently than those from male participants into the ‘Experiences and Consequences of Attraction and Interest’ category (*χ*^2^ = 11.236 (1, *n* = 571), *p* < 0.001, *ϕ* = 0.140), with small effect sizes. All comparisons are presented in Table 4. Overall, it seems that gender and sexual identities were not associated with the content of participants’ romantic chemistry conceptualisations and the strength of the only association observed was small.

## 4. Discussion

Our findings extended upon previous understandings of lay conceptualisations of romantic chemistry using qualitative data and content analysis methodologies. Many of our categories align with the themes constructed by Campbell et al. (2018), although our categories attempted to capture more of the complexity within the aspects common between studies. For example, Campbell and colleagues’ ‘Similarities’ theme is expanded and complicated in our ‘Feelings of Connection and Alignment’ category. This category was much more prominent in our study, being coded into 25% of responses, whereas Campbell and colleagues only coded ‘Similarities’ into approximately 5% of responses in their study. We made decisions to consolidate categories that Campbell and colleagues may not have, such as content fitting their ‘Reciprocal Candor’ and ‘Mutual Enjoyment’ themes into our ‘Comfortable and Genuine Interactivity’ category. This enabled us to focus our own ‘Mutual Feelings’ category on instances of generally shared feelings, as opposed to only those of enjoyment which we believed fit more within our interactivity category. Overall, we argue that our categories replicate and extend upon the themes of Campbell and colleagues’ study, while also highlighting important differentiations in areas of connection and attraction.

The categories we constructed through our analysis align well with the process model proposed by Reis et al. (2022). The three broad areas of interaction, connection, and attraction appear to respectively map onto Reis and colleagues’ behavioural, cognitive, and affective components of perceived chemistry. Reis and colleagues do not suggest these components exist in isolation and we agree; there was a considerable combination of these categories within many responses. We note that participants’ experiences of interaction, connection, and attraction could manifest in behavioural, cognitive, or affective ways, which we attribute to romantic chemistry being a perceived experience and open to individual interpretation. We captured these ideas in our ‘Multifaceted or complicated’ category, where the complicated elements of the category seem to, in part, reflect participants attempts to understand the multifaceted nature of romantic chemistry.

We speculate that the multifaceted nature of romantic chemistry could contribute to difficulties experienced in research whereby the complicated elements interfere with conceptualisation, measurement, and prediction. For example, when participants respond to a question that asks them to rate the level of romantic chemistry, what are they considering, and do current measures of chemistry sufficiently capture the dimensions and variance seen in conceptualisations of romantic chemistry? Current measures of romantic chemistry are not psychometrically validated as they often rely on single items or inferred through a combination of items that theoretically contribute towards chemistry perceptions (Eastwick et al., 2007; Baxter et al., 2022). This does not mean that these tasks are not possible; for example, it might be appropriate to use factor analysis to explore the dimensionality of romantic chemistry aspects to better measure perceptions. If such dimensionality was established, researchers could explore if circumstances where individuals appear to only be perceiving or experiencing romantic chemistry on one dimension result in weaker levels of romantic interest (Eastwick et al., 2007). We hope our findings assist future researchers to address the complexity of romantic chemistry through this suggestion of factor analysis and other directions.

The proportion of responses we coded into interaction, connection, and attraction categories were similar when considering unique responses across each category and respective sub-categories. Despite this similarity, the ‘Comfortable and Authentic Interactivity’ category is distinct in the absence of a sub-category. While some connection and attraction responses were precise enough to form sub-categories, most interaction responses were concerned with conversation or being content in the absence of conversation. A reoccurring theme in interaction responses was humour and laughter, which have been observed to be important for relationships generally (Kurtz & Algoe, 2015), but these phenomena also contributed to experiences of connection and attraction for participants. We considered a separate category that captured humour and laughter content but determined that the meanings behind this category would not be distinguishable from those of our interactivity category. This does not mean humour and laughter should be amalgamated into interactivity and diminished; in fact, we believe that humour and laughter are important enough to be the focus of future research, as these phenomena consistently appear in romantic research but are infrequently studied (Devenport et al., 2025; Li et al., 2009; Wainwright et al., 2024). Although humour resulted in interactivity outcomes, the contributions to connection and attraction may indicate that, similar to romantic chemistry, there is scope to explore the dimensionality of humour within romantic contexts, perhaps in focused qualitative or factor analysis investigations.

Many participants expressed conceptualisations of instantaneous romantic chemistry connections that are usually the most salient in previous understandings of romantic chemistry (Campbell et al., 2018; Shen & Qian, 2024). While our findings support the importance of these quick connections nonetheless, a noticeable number of participants focused on slower-to-form, deeper connections. There were responses where both forms of connection were mentioned and some participants contrasted the forms to express their learned caution around instantaneous connections, which were not necessarily indicative of long-term potential. This expression aligns with findings that deeper understanding and appreciation improve relationships (Gordon & Diamond, 2023) and professional romantic matchmakers who suggest romantic chemistry cannot be beneficial if it is instantaneous (Sharabi, 2024). We do not believe this means that all instantaneous romantic chemistry is inherently detrimental, just that the quick nature of these connections, and the associated intensely positive feelings, may lead individuals to neglect critical reflection on the event. This finding could be linked to the debate about implicit and explicit processing in romantic partner selection (Eastwick et al., 2011), whereby implicit, affective processing (e.g., on a date) is more predictive of partner selections than explicit, abstract processing (e.g., reviewing online dating profiles). We must necessarily limit our speculation on how forms of connection interact with processing styles, but future research could potentially explore these ideas by tracking the trajectories of romantic relationships that begin with instantaneous connections (Eastwick et al., 2019).

While our sample of single participants actively looking for a romantic partner permits speculation about romantic partner selection, romantic chemistry is also strongly linked to love and attachment processes (Reis et al., 2022). Romantic partner selection literature is dominated by evolutionary perspectives, where romantic chemistry may be framed as a functionally adaptative neurochemical system of lust, attraction, and attachment (Fisher et al., 2002; Sorokowski et al., 2021). These ideas have apparent equivalents to our categories of attraction, desire, and connection but may not readily encompass our interactivity category. Comfortable and genuine interactivity may have increased importance for those seeking a romantic partner and actively dating. Within the romantic partner selection context, these interactions may enable perceptions of romantic chemistry and be the beginning of pathways to deeper feelings of love and attachment. Of course, comfort and genuineness could also be theoretically tied to trust, safety, and security, which are important evolutionary needs and theorised components of chemistry (Reis et al., 2022). The various constructs associated with romantic chemistry may vary slightly during the early relationship formation phase, including romantic partner selection, but further research is needed to further delineate phases and differences therein.

Experiences of intellectual attraction were not as frequently expressed as other aspects of romantic chemistry but contribute to the growing literature that highlights the importance of intellectual elements in romantic contexts (Gignac et al., 2018; Rossignac-Milon & Higgins, 2018). We labelled these experiences as ‘attraction’ phenomena due to language that implied desire for more of a prospective partner, whereas mental or intellectual ‘connection’ phenomena were more descriptive of similarity and alignment. It is entirely possible that intellectual elements are associated with the same outcomes regardless of attraction or connection distinctions. For example, Rossignac-Milon and Higgins (2018) frames shared feelings and evaluations as a way that individuals connect intellectually via establishment of a shared reality, which has also been linked to relationship initiation when paired with authenticity (Rossignac-Milon et al., 2024). Our findings suggest that intellectual attraction could act similarly; where intellectual challenge is desirable and perceived positively, individuals may be attracted while simultaneously moving towards a shared reality. Intellectual attraction also fits within the contemporary concept of sapiosexuality that describes individuals who are attracted to romantic or sexual partners based primarily on perceptions of intelligence (Gignac et al., 2018). The nature of intellectual connection and attraction in romantic contexts need to be investigated further and would suit qualitative research specifically focused on these phenomena, perhaps with sapiosexual populations.

A large body of quantitative literature suggests that males prioritise attraction to prospective romantic partners, particularly physical attraction (Buss et al., 2020; Devenport et al., 2025; Thomas et al., 2020). We observed the opposite of this assigned-sex difference in our deductive analyses, as female participant responses were coded into the general attraction category significantly more often. We acknowledge the limited nature of this finding as it is based on our deductive analysis of qualitative responses into our inductively constructed categories, but this finding does align with previous research (Campbell et al., 2018). It is important to note that the quantitative literature cited focuses on preference while this study focused on perceptions of romantic chemistry. Male participants preference for attraction may mean that they have attraction experiences more often, which may diminish the novelty of that experience and limit their ability to perceive it as ‘special’ enough to contribute to romantic chemistry (Reis et al., 2022). A corollary of this possibility would be that female participants’ lower preference for attraction means that they have attraction experiences less often, and thus these experiences are perceived as more ‘special’ and contribute more to their perceptions of romantic chemistry. Although we believe our speculation is theoretically sound (Reis et al., 2022), there is a need to investigate the possibility that novel, positive experiences of attraction, and interaction and connection, contribute more strongly to perceptions of romantic chemistry, perhaps by exploring associations between perceptions of positive novelty and romantic chemistry. It is important to note that this was the only significant difference based on assigned-sex, gender, or sexual identity, which may indicate that romantic chemistry is a universal experience with individual perceptions shaped by preference or experience rather than identity.

While performing inductive analyses on qualitative data sourced from survey responses can be limiting, we found participants’ responses to be sufficiently rich for our analyses. We could not avoid participants’ conceptualisations of romantic chemistry experiences being provided retroactively. This limitation was somewhat managed by recruiting only those who were currently looking for a romantic partner. This meant that participant responses were more likely to be based on recent experience and less likely to be based on a current relationship, which may have caused responses to be representative of chemistry within a specific relationship instead of their general conceptualisation. The question asked to participants could have been expressed more clearly, including placing the words ‘romantic’ and ‘chemistry’ together, but participants responses do not indicate there was a systematic misunderstanding of the concept or question.

The need for qualitative data that is collected as close as possible to romantic chemistry experiences persists (Campbell et al., 2018) and may be possible through methods such as ecological momentary assessment (Wrzus & Neubauer, 2023). This kind of data may be richer than the data in this study and may enable the use of deeper qualitative methodologies such as grounded theory approaches (Charmaz, 2006), which could generate stronger theoretical understandings of romantic chemistry. Furthermore, the influence of age differences on experiences and conceptualisations of romantic chemistry was not a focus of the study but could be relevant due to differences in preference (Driebe et al., 2024) and experiences of emotion (Burr et al., 2021). Our sample was quite diverse in age, but future research may benefit from exploring romantic chemistry in age-limited samples and could also consider cultural and cross-cultural contextual influences (Karandashev, 2025) as our sample was potentially culturally homogenous.

Our study provides necessary clarification regarding lay conceptualisations of romantic chemistry, which aligned with current theory, and should enable future research to approach romantic chemistry with increased knowledge. Although theory and our findings suggest that chemistry is multifaceted, this had not been sufficiently addressed in prior qualitative research. Participant responses consistently suggested that this multifaceted nature meant that romantic chemistry required a combination of comfortable interaction, meaningful connection, and felt attraction to eventuate. We identified multiple elements of romantic chemistry that warrant focus in future research, including humour and laughter, intellectual attraction and connection, and the contribution of perceived novelty to perceptions of romantic chemistry. We hope that our findings will assist future researchers to harness lay understandings of romantic chemistry in the development of their conceptualisations, and aid in the work of measuring, predicting, and investigating the role of romantic chemistry in various contexts.

## Figures and Tables

**Table 1:** Final sample minority gender and sexual identities proportions (*n* = 303).

Identity Group	*n*	% of Full Sample
Gender identity		
Transman	15	2.62%
Transwoman	12	2.10%
Non-binary	47	8.23%
Sexual identity ^a^		
Gay	65	11.38%
Lesbian	33	5.78%
Bisexual	120	21.02%
Pansexual	55	9.63%
Asexual	47	8.23%
Polyamorous	41	7.18%

*Note.* ^a^ Participants could identify as one of gay, lesbian, bisexual, and pansexual. Asexual and polyamorous identifications could be made alongside other identities.

**Table 2:** Final categories and sub-categories capturing participants’ conceptualisations of romantic chemistry.

Category/Sub-Category	Category/Sub-Category Rationale	Number of Responses Deductively Coded into Category/ Sub-Category	Proportion of Responses Deductively Coded into Category/ Sub-Category
Multifaceted or Complicated	Captured responses suggesting that chemistry required multiple components to be present simultaneously, which was frequently confusing and/or special to experience	287	50.26%
The Importance of Mutual Feelings	Captured responses highlighting that chemistry was mutually felt and the necessity of this mutuality for chemistry to be real	114	19.96%
Comfortable and Genuine Interactivity	Captured responses that focused on chemistry involving interactions between parties that felt simple, relaxing, and open	306	57.44%
Feelings of Connection and Alignment	Captured responses that identified chemistry in a way that involved the parties having a similarity and/or matching in some manner and did not fit within sub-categories	146	25.57%
Energetic and Instantaneous Connections	Captured responses which expressed that chemistry involved a connection that was quick, uncontrollable, and had a ‘spark’	85	14.89%
Deep and Emotional Connections	Captured responses which expressed that chemistry involved a connection that was developed over time through depth, understanding, and feelings	70	12.26%
Experiences and Consequences of Attraction and Interest	Captured responses that identified chemistry in a way that involved the individual or both parties feeling drawn to the other party and/or the consequences of such feelings and did not fit within sub-categories	200	36.78%
Experiencing and Expressing Physical and Sexual Attraction	Captured responses which expressed that chemistry involved physical elements such as bodily sensations, lustful feelings, and physical arousal	143	25.04%
Experiencing Intellectual Attraction	Captured responses which expressed that chemistry involved intellectual elements such as views being challenged, open discussions, and holding similar opinions	46	8.06%

**Table 3:** Categories, subcategories, and associated quotes with proportion of responses coded into each category and subcategory.

**Multifaceted or Complicated**	**The Importance of Mutual Feelings**	**Comfortable and Authentic Interactivity**
Chemistry is the ability to click with another person on an intellectual, emotional or physical level. Having all 3 accounts for a good relationship. It’s mostly indefinable but means you “get along” and have a mutual attraction that is physical, sexual, and possibly emotional but not always. Chemistry is foremost to me having a similar sense of humour. There is no chemistry if you aren’t laughing. After that it’s a combination of physical attraction and similar values. I believe chemistry has a lot to do with connection. When I was younger it was more about sexual chemistry or attraction but after considering my attachment style, I go a lot less for that crazy chemistry at first and see how good attraction and commonalities go, over time. Difficult to explain—some sort of connection and being on the same wavelength, but also a feeling of more than friendship at the time. It’s a spark between people, either you feel it, or you don’t. Eye contact, smiling, banter, mirrored body language, a level of comfort and ease in interacting.	Feeling that we are both able to relax and be ourselves without too much exposition, combined with a base level of attraction on both of our parts. When you both know you’re into each other, and you’re having fun, and you don’t have to say it (you might, to be funny, but you don’t have to). Where you just get along with someone, can have easy and free conversation that feels comfortable. When you feel sexually attracted to them and can sense that they feel the same way about you. It’s a combination of—someone whom I’m attracted to and someone who clearly is attracted to me (could be subtle or obvious) +/- someone I develop a connection with. Possibly, good conversation, discovered shared interest, or similar sense of humour (sometimes mutual attraction is enough to feel like chemistry, for better or worse). Chemistry for me, is when you both are able to effortlessly build each other up to be the best versions of each other, when you both feel that glow from within after seeing each other. I believe it is the mutually shared feeling between two consenting adults of a connection or personal affinity for one another.	Having the conversation flow and not be so question orientated, lots of laughter, the ability to easily be open, it feels safe. Chemistry to me is when conversation flows easily between you and your romantic partner. some people would describe chemistry as that feeling of butterflies in your stomach, but I actually believe it is the opposite of that. chemistry should feel like comfort, it should feel like you’re happy to see your partner without any nervousness. Finding myself drawn into a conversation & everything just seems to flow, time disappears & I don’t think about whatever else is happening, only the now. A feeling of ‘bouncing off’ of one another. Feeling comfortable to be genuine and open. Easily flowing conversation. A feeling of comfort or ease around a particular person where you can genuinely consider yourself safe, especially if you were to be vulnerable. Conversations that flow easily—Light humour occasionally—When there is genuine interest in each other’s stories (e.g., asking questions and responding to answers to questions genuinely and openly).
**Feelings of Connection and Alignment**	**Energetic and Instantaneous Connections**	**Deep and Emotional Connections**
Connection, a sense of shared sense of outlook. Feeling that you and the other person have seen the world through a similar lens. Having similar mindsets (though not necessarily the same opinions), how easy it is to talk to and understand them, having a mix of similar and different but complementary personality traits, finding each other to be interesting people. Chemistry is how compatible your lifestyle is with other partners, and how that works for your preferences. Chemistry is being able to understand each other, immediately getting jokes and wanting a lot of (not necessarily all) the same things in life. A common thing between both parties and working in harmony. To click—appreciate each other’s sense of humour—have complementary lifestyles—to laugh at the same thing (similar sense of humour) sexual attraction. It is a connection of mind and body. When you “click” with someone, and it feels effortless.	It’s the spark, the natural energy existing between two individuals. It’s magnetism. I had fell in love with a guy in my early twenties. When we first laid eyes on each other it was love at first sight. It really blew my socks off. We couldn’t stop looking at each other, he gave me butterflies in my stomach. A feeling of electricity between two people, a “spark”, a feeling like you’ve known them forever. It makes the person seem to glow bright and if the chemistry is sexual rather than simply romantic then “sparks fly”. (I have synaesthesia, so for me this comment is literal, when there is chemistry “sparks” really do “fly”). And with this, they smell good. I’m not sure. I suppose chemistry is something spontaneous and uncontrollable—like when you barely know each other but just mesh really well and have this instant connection. As soon as you meet something clicks and you immediately feel like you’ve known them your whole life. Or even if that feeling builds up over time, you feel like you can both keep talking for hours.	Connecting with someone on an emotional level that is stronger than a “normal” friendship. It’s the emotional connection between the two of you. You might have different beliefs and views in life but when you start to agree to disagree you meet halfway to make it work. Chemistry for me means that the two people can connect with each other at a deeper, meaningful level combined with romance and intimacy. Being demisexual, I get to know someone and if there’s an emotional connection I go from there. Chemistry is if I feel a deep connection with another person and am on a similar or complementary wavelength. We connect on an emotional level and feel deeply about similar issues. I click with them and feel deeply connected with them as a person. Chemistry for me means that these feelings are reciprocated, and I feel appreciated by them (rather than insecure that they don’t feel the same way). Understanding someone’s personality, like being able to predict their mood, thoughts and enjoying their sense of humour.
**Experiences and Consequences of Attraction and Interest**	**Experiencing and Expressing Physical** **and Sexual Attraction**	**Experiencing Intellectual Attraction**
A sense of feeling captivated and intrigued to learn more about each other, cheeky and flowing conversation. A feeling of wanting to be closer to someone, curiosity is high, my body seems to want more. I feel happier in their presence. This doesn’t have to be strongly sexual, though would often lead my thoughts there over a period of time. Insatiable attraction to someone’s body and mind; you can’t get enough of them. Often, a sense of mutual interest/attraction, flirting, similar “love language”. A deep desire to see that person all the time. A situation where you’re interested in them and no one else, where you feel drawn to someone and their demeanour. Feeling drawn to the other person, wanting to know everything about them, and not wanting the conversation to end.	When you downright want to fuck in the craziest way no matter where you are. Someone who physically arouses you and who you want to touch and be physically with all the time. Chemistry is a physical connection/desire that is shared between both parties. Wanting to be close or share any level of touch (e.g., holding hands). Not just sexual desire but a desire to be more intimate with the other person in any way. Chemistry is not wanting to keep my hands off of them/wanting to kiss them all the time. I like a man’s smell, firmness and especially, large hands, and big arms. :-) Smile, humour and deep resonance in his voice definitely works too. A physical response, strong attraction. The desire to be physically close to a person. Physical contact sets your heart racing and your skin tingles with anticipation/excitement. It’s like a smell… it almost feels like you vibrate at the same time… it’s intoxicating.	Someone who can take my breath away. Someone not only I am attracted to physically but mentally too. A strong intellectual attraction. Jokes and laughter are essential for me to feel “chemistry” with another person, and I feel most admiration and excitement when someone can challenge me intellectually and can teach me new things. If their vibe and my vibe clicks very well, a lot of common grounds and common interest. They are different but their difference is able to make me grow and wonder philosophical stuff. Being able to talk about any subject, continual banter that challenges my thoughts or beliefs. Encouraging new thought processes. Being able to have frank honest discussions and believing that what is said is what is meant with no hidden motivation or implied intent. A similar sense of humour. How easy it is to be around them. How comfortable silences are between us. Similar opinions on social justice/societal issues.

**Table 4:** Proportions of responses within each subsample compared in deductive analyses.

Category or Sub-Category	Male	Female	Straight	Gay/Lesbian	Bisexual/Pansexual	Cisgender	Trans and Gender Diverse	Majority Identity	Minority Identity
Multifaceted or Complicated	45.02% (*n* = 104)	53.82% (*n* = 183)	47.57% (*n* = 54)	57.14% (*n* = 56)	51.72% (*n* = 90)	50.10% (*n* = 248)	51.32% (*n* = 39)	47.76% (*n* = 128)	52.48% (*n* = 159)
The Importance of Mutual Feelings	23.38% (*n* = 54)	17.65% (*n* = 60)	21.88% (*n* = 63)	22.45% (*n* = 22)	16.67% (*n* = 29)	20.20% (*n* = 100)	18.42% (*n* = 14)	21.27% (*n* = 57)	18.81% (*n* = 57)
Comfortable and Genuine Interactivity	60.61% (*n* = 140)	57.64% (*n* = 166)	57.64% (*n* = 166)	55.10% (*n* = 54)	58.05% (*n* = 101)	57.58% (*n* = 285)	56.58% (*n* = 43)	47.76% (*n* = 128)	23.43% (*n* = 71)
Feelings of Connection and Alignment	23.38% (*n* = 54)	27.06% (*n* = 92)	27.08% (*n* = 78)	23.47% (*n* = 33)	23.56% (*n* = 41)	24.85% (*n* = 123)	30.26% (*n* = 23)	27.99% (*n* = 75)	16.83% (*n* = 51)
Energetic and Instantaneous Connections	12.99% (*n* = 30)	16.18% (*n* = 55)	12.85% (*n* = 37)	21.43% (*n* = 21)	15.52% (*n* = 27)	14.75% (*n* = 73)	15.79% (*n* = 12)	12.69% (*n* = 34)	13.53% (*n* = 41)
Deep and Emotional Connections	12.55% (*n* = 29)	12.06% (*n* = 41)	10.42% (*n* = 30)	12.24% (*n* = 12)	14.37% (*n* = 25)	11.31% (*n* = 56)	18.42% (*n* = 14)	10.82% (*n* = 29)	38.61% (*n* = 117)
Experiences and Consequences of Attraction and Interest	**28.57%** **(*n* = 66)**	**42.35%** **(*n* = 144)**	34.72% (*n* = 100)	34.69% (*n* = 34)	41.95% (*n* = 73)	36.16% (*n* = 179)	40.79% (*n* = 31)	34.70% (*n* = 93)	38.61% (*n* = 117)
Experiencing and Expressing Physical and Sexual Attraction	21.21% (*n* = 49)	27.65% (*n* = 94)	25.00% (*n* = 72)	27.55% (*n* = 27)	24.71% (*n* = 43)	24.85% (*n* = 123)	26.32% (*n* = 20)	25.37% (*n* = 68)	24.75% (*n* = 75)
Experiencing Intellectual Attraction	6.06% (*n* = 14)	9.41% (*n* = 32)	5.56% (*n* = 16)	7.14% (*n* = 7)	12.07% (*n* = 21)	8.28% (*n* = 41)	6.58% (*n* = 5)	5.60% (*n* = 15)	10.23% (*n* = 31)

*Note*. The bolded comparison was the only significantly different result at the alpha level adjusted for multiple comparisons (0.006).

## Data Availability

It was not possible to share de-identified data as ethical approval for the study did not provide for sharing of data publicly and participants only consented to data being shared with and analysed by the research team.
